# The inheritance of pathogenic mitochondrial DNA mutations

**DOI:** 10.1016/j.bbadis.2009.03.002

**Published:** 2009-12

**Authors:** L.M. Cree, D.C. Samuels, P.F. Chinnery

**Affiliations:** a Mitochondrial Research Group, Institute for Ageing and Health, Newcastle University, UK; b The Center for Human Genetics Research, Department of Molecular Physiology and Biophysics, Vanderbilt University Medical Center, USA

**Keywords:** mtDNA, Mitochondria, Genetic bottleneck, Inheritance

## Abstract

Mitochondrial DNA mutations cause disease in > 1 in 5000 of the population, and ∼ 1 in 200 of the population are asymptomatic carriers of a pathogenic mtDNA mutation. Many patients with these pathogenic mtDNA mutations present with a progressive, disabling neurological syndrome that leads to major disability and premature death. There is currently no effective treatment for mitochondrial disorders, placing great emphasis on preventing the transmission of these diseases. An empiric approach can be used to guide genetic counseling for common mtDNA mutations, but many families transmit rare or unique molecular defects. There is therefore a pressing need to develop techniques to prevent transmission based on a solid understanding of the biological mechanisms. Several recent studies have cast new light on the genetics and cell biology of mtDNA inheritance, but these studies have also raised new controversies. Here we compare and contrast these findings and discuss their relevance for the transmission of human mtDNA diseases.

## 1 Introduction

Recent epidemiological studies confirm that pathogenic mitochondrial DNA (mtDNA) mutations are a major cause of human disease, affecting at least 1 in 5000 of the population [1,2]. Pathogenic alleles are present in > 1 in 200 live births [3], and occur *de novo* at least every 1000 births [3]. Many mtDNA mutations are transmitted down the maternal line and cause progressive, disabling multi-system disease, often with devastating effects on the nervous system. Treatment options are limited [4], and focus on the management of complications [5]. These facts place great emphasis on the development of techniques to prevent transmission in the future. This review will focus on recent advances in our understanding of the biological basis of mtDNA transmission, highlighting the importance of this work for human pedigrees transmitting pathogenic mtDNA mutations. The major focus of this review is on the underlying basic scientific principles, so we will not discuss clinical techniques under development to prevent transmission through nuclear transfer, nor methods being used for implantation and pre-natal diagnosis (for a consideration of these topics, the reader is referred to several recent reviews [6–8]). This article also assumes a basic understanding of mtDNA mutations and human disease which can be obtained from other articles in this *Special Issue* of *Biochemica et Biophysica Acta* on *Mitochondrial Diseases*.

## 2 Are all pathogenic mtDNA mutations maternally inherited?

MtDNA is almost exclusively maternally inherited in mammals [9], probably because sperm mtDNA is tagged with ubiquitin and actively degraded in the early pre-implantation embryo [10]. A single case of paternal transmission of a pathogenic mtDNA mutation has been described in a patient with a myopathy [11], but has not been noted in any other cases despite systematic investigation [12,13]. Paternal transmission of mtDNA is therefore likely to be an extremely rare event in humans. In practical terms, this means that men with mtDNA disease cannot pass the disorder on to their offspring.

On the other hand, women harbouring homoplasmic mtDNA mutations can transmit mutated mtDNA to their children. Homoplasmic mutations are typically associated with a mild biochemical phenotype, and cause organ-specific mitochondrial disease. For example, the three point mutations (11778G > A, 14484T > C; 3460G > A) in complex I (*MTND*) genes that cause Leber hereditary optic neuropathy (LHON) preferentially target the retinal ganglion cell and cause blindness [14]; and the 1555A > G mtDNA 12S rRNA gene mutation causes isolated sensori-neural deafness [15]. Intriguingly, in both cases the phenotypic segregation pattern implicates additional factors in the pathophysiology, including environmental triggers and interacting nuclear genetic loci [16–18]. However, in both cases it is possible to determine recurrence risks empirically by observing many pedigrees [19–21]. With the exception of these two examples, homoplasmic pathogenic mtDNA mutations have probably been under-recognised in the past, partly due to difficulties in proving that a genetic variant is pathogenic and not simply a population polymorphism [22,23].

By contrast, many pathogenic mtDNA mutations are heteroplasmic, with affected individuals harbouring varying proportions of mutated and wild-type mtDNA [24]. Given that overall mutation load broadly correlates with the clinical phenotype [25,26], the difference in the inherited mutation load partially explains the clinical variation between siblings in the same family [26–28]. Understanding the mechanism of inheritance of heteroplasmic mtDNA mutations is a major challenge facing clinicians and scientists working in the field.

For some pathogenic heteroplasmic mtDNA mutations, the empiric recurrence risks are low. This led to the previously widely held view that mtDNA deletions were not transmitted from affected women to their offspring. Although disease recurrence was observed in some families, it was thought that related mtDNA duplications were the actual transmitted molecule, leading to deletion formation in the offspring [29,30]. However, the detection of an identical deletion in one mother and child [31] strongly suggests that the deletions themselves can be transmitted, albeit rarely. A systematic investigation of over 200 pedigrees estimated the recurrence risk at ∼ 1 in 24 [32]. There are also a number of rare muscle-specific mtDNA point mutations which also do not appear to be transmitted [33–36]. Although these apparently sporadic cases could all be due to somatic mutations that do not involve the germ line, an alternative view is that these mutations are at one extreme of a spectrum, where there is selection against the transmission of mutated mtDNA. Understanding the mechanisms involved provides the key to future prevention strategies.

## 3 Heteroplasmic pathogenic mtDNA mutations in human pedigrees

Women harbouring heteroplasmic mtDNA mutation can transmit a wide range of heteroplasmy levels to different offspring within the same sibship. In one meta-analysis of 338 transmitted point mutations, for some mutations the percentage level of mutant mtDNA tended to increase with transmission, and for others the level seemed to decrease [37]. However, these data were collected retrospectively through an affected individual, and the apparent differences could be due to ascertainment bias [37]. Recent work suggests there may be selection against 3243A > G during transmission, and that shifts in heteroplasmy might depend on the level in the mother [38]. Again, these findings are difficult to interpret because the level of heteroplasmy is often markedly different between different tissues and organs, and the 3243A > G mutation level in blood decreases exponentially throughout life [39–41]. For some mutations, the level of heteroplasmy measured in blood is therefore unlikely to reflect the level in the ovary. On the other hand, for some mtDNA mutations (e.g. 8993T > G/C), the level of heteroplasmy is remarkably consistent in different tissues, and does not change during life [42]. Under these circumstances, it may be possible to reliably study transmission by measuring heteroplasmy values in blood from different family members [26].

Intriguingly, the change in heteroplasmy seen during transmission does appear to differ between mutations. Rapid shifts are commonplace in families transmitting 8993T > G/C [26], but less likely in families transmitting 8344A > G [43]. This explains why 8993T > G/C pedigrees tend to be small, because the deleterious mutation is either lost from the maternal line, or reaches very high levels and causes severe disease in childhood, thus preventing further transmission [26]. On the other hand, 8344A > G pedigrees are often large, and span many generations [43]. This anecdotal evidence implies differences in the mode of transmission between different genetic variants of mtDNA. Further advances in our understanding of the mechanisms involved have been revealed by studying the transmission of ostensibly neutral mtDNA heteroplasmy in other mammalian species.

## 4 Inheritance of mtDNA heteroplasmy and the genetic bottleneck

By studying the heteroplasmy levels of neutral mtDNA sequence variants in the blood of a female founder Holstein cow and her offspring, Hauswirth and Laipis showed that the allele frequency of these variants rapidly shifted and became fixed in a few generations [44]. These results led to the proposal of the mitochondrial genetic bottleneck hypothesis whereby a decrease in the number of mitochondrial genomes repopulating the offspring of the next generation causes a sampling effect during transmission, leading to the rapid changes in heteroplasmy during one generation. The presence of a mitochondrial genetic bottleneck gained support from studies of heteroplasmic mice transmitting neutral mtDNA polymorphisms. By studying the progeny of mice carrying apparently neutral BALB/c and NZB mtDNA sequence variants, Jenuth et al. found that the offspring of a single heteroplasmic mother can have differing levels of heteroplasmy [45]. The mean level of heteroplasmy amongst all of the offspring was approximately equal to the heteroplasmy level seen in the mother, implicating mechanism governed by random genetic drift [45]. A recent analysis (cite) of this and other human and animal model data confirms that the heteroplasmy levels measured in large numbers of cells follow a distribution from theoretical population genetics based on random genetic drift [48].

Primordial germ cells (PGCs) are the first committed germ-line precursor cells in mammals, which usually appear around day 7.25 post conception in the mouse, and day 20 in the human, at the base of the allantois. By measuring the proportion of BALB/c and NZB mtDNA sequence variants in mature oocytes, primary oocytes and PGCs, Jenuth et al. also found that nearly all of the genotypic variance was generated during the development of the female germ line, and before the primary oocyte population had been formed. They concluded, therefore, that the mtDNA bottleneck is occurring at the very early stages of oogenesis and that for these neutral sequence variants at least, the transmission of heteroplasmy to the offspring is largely governed by random genetic drift. The authors estimated the number of segregating units (i.e. the size of the mouse mtDNA bottleneck) using an adaptation of the population genetic model of Sewell-Wright [46], assuming that the genetic bottleneck was fixed over 15 cell divisions. They predicted a germ-line bottleneck of 185 mtDNA molecules in the mouse was sufficient to explain the segregation of BALB/NZB mtDNA heteroplasmy [45].

Evidence that the same mechanism operates in humans transmitting pathogenic mtDNA mutations came from the study of 82 single primary oocytes from a woman carrying the 3243A > G mtDNA mutation [47]. The frequency distribution of oocyte mutation load corresponded to a binomial distribution (although recent analysis suggests a slightly different distribution provides a more accurate description [48]). Equal numbers of oocytes had mutation levels greater or less than the mean, in keeping with the random genetic drift mechanism, and with the same population genetics model [46] (adapted for humans, with 23 cell divisions), the bottleneck was estimated as 173 segregating units [45,47].

## 5 Drift or selection?

Recent evidence has cast light on this key issue. Stewart et al. studied the transmission of mtDNA mutations introduced into the mtDNA mutator mice due to the expression of a proofreading deficient mtDNA polymerase [49]. Female offspring of these mutator mice, which carried approximately 30 mutations randomly distributed throughout the mitochondrial genome, were continuously backcrossed to wild-type males to remove the mutant *POLG* gene but allow the transmission of the heteroplasmic mtDNA mutations [50]. These heteroplasmic mtDNA mutations were then followed through 6 successive generations. Results from this study show that there is a rapid loss of non-synonymous (amino-acid changing) mutations in the protein coding genes as early as the second generation when compared to synonymous (silent) changes. This suggests strong purifying selection against deleterious mtDNA mutations in the mouse germ line. Interestingly the authors also noted an excess of mutations in some genes (*MTCYB*, *MTATP6*, *MTATP8*), which is consistent with findings in humans where there appear to be a large number of *de novo* mutations especially in the ATP6 gene.

Using a different experimental strategy, Fan et al. also found evidence for purifying selection by studying the transmission of a *MTND6* frameshift mutation which inactivates oxidative phosphorylation complex I when homoplasmic [51]. The *MTND6* mutation was eliminated from the mouse female germ line within four generations. In contrast, a milder mis-sense mutation in the cytochrome *c* oxidase subunit I gene (*MTCOI*), which reduced complex IV activity by 50% when homoplasmic, was repeatedly transmitted over successive generations despite causing mitochondrial myopathy and a cardiac phenotype [51]. Taken together with the study from Stewart et al. these results provide evidence for selection of some mtDNA mutations in the mouse female germ line and suggest that these severe mtDNA mutations encoding non-synonymous changes are selected against at the organelle level. However, the mechanism may not be straight-forward, with some deleterious mutations being selected against (purifying selection), and some possibly undergoing positive section [24].

## 6 How is mtDNA inherited?

Recent technological advances have enabled three groups to directly test the bottleneck hypothesis by measuring mtDNA in single cells during early development [52–54]. *Stella* is one of the first proteins expressed by the committed germ line [55]. By studying pre-implantation mouse embryos and *Stella*-GFP transgenic mice to unambiguously identify PGCs using quantitative PCR, we showed that the amount of mtDNA within individual PGCs has a median value of 203 molecules at 7.5 days post conception (dpc) [53]. These first discernable PGCs contained substantially lower mtDNA copy numbers than that of the individual blastomeres making up pre-implantation embryos. Following implantation, we showed that the mtDNA copy number in the PGCs steadily increased as the cells migrate to the genital ridge, reaching ∼ 1500 copies in each PGC by 14.5 dpc, however this is still almost 100 fold lower than the level seen in the mature oocyte. Using this experimental data to model the segregation and replication of mtDNA between dividing cell in the germ line, we showed that the mtDNA copy numbers that we measured could cause a genetic bottleneck which explains the genotypic variance seen in the offspring of 246 offspring from 22 litters born to mothers with different levels of NZB/C57Bl.6J heteroplasmy. This work study suggests ∼ 70% of the heteroplasmy variance is generated due to the unequal portioning of mtDNA molecules into daughter cells during both pre- and post-implantation development, when the amount of mtDNA molecules within the cell drops to a median value of approximately 200. The remaining ∼ 30% variance being generated during the mitotic expansion of the PGC population, where copy number values approximate 1500 molecules [53]. However, direct measurements of heteroplasmy were not made at each stage in development in these experiments.

In an earlier study, Cao et al. also estimated mtDNA copy number in mature oocytes, pre-implantation embryos and PGCs [52]. Whilst copy number values in mature oocytes and pre-implantation embryos were similar to both our own data and those of others [53,56], the average value in day 7.5 PGCs was ∼ 1700 copies, which was significantly higher than the 200 copies we measured at the same time point. Intriguingly, Cao et al. did not report any difference in the mtDNA copy number between 7.5 dpc and 13.5 dpc and concluded that the mtDNA bottleneck was not due to a decrease in mtDNA copy number in early oogenesis, rather that it is due to a small effective number of segregating units, each containing several mtDNA molecules [52]. Differences in methodologies may explain these apparent inconsistencies. Cao et al. used alkaline phosphatase histochemistry to identify the primordial germ cells in contrast to the *Stella*-GFP transgenic mouse used in our study. In our hands real-time PCR traces strongly suggested that the alkaline phosphatase stained cells inhibited the real-time PCR reaction in a stochastic manner, increasing the variability between measurements and potentially compromising the result.

It is also possible that the two studies focused on slightly different developmental stages. At any given time there is considerable variation in the developmental stage of individual mouse embryos [57]. This is partly due to subtle variations in the precise time of conception, which cannot be directly controlled. For example, at 7.5 dpc, embryos range between the mid-streak stage and the early head-fold stage even within the same litter [57]. PGCs appear from the early allantoic bud neural plate stage. Cao et al. identified and studied late allantoic bud stage embryos. By contrast, using flow cytometry and a highly-specific PGC marker, we studied both early and late allantoic bud stage embryos [53]. In keeping with this, the range of mtDNA copy number values at 7.5 dpc reported by Cao et al. (1258 to 3335) fell within the upper end of the range of values we reported (26 to 3402) [53]. The lower copy numbers that we measured could therefore have been from early allantoic bud stage embryos, which Cao et al. specifically did not study. This suggests that the early allantoic bud stage is the time point when the amount of mtDNA within individual PGCs falls to its lowest level.

**Fig. 1:**
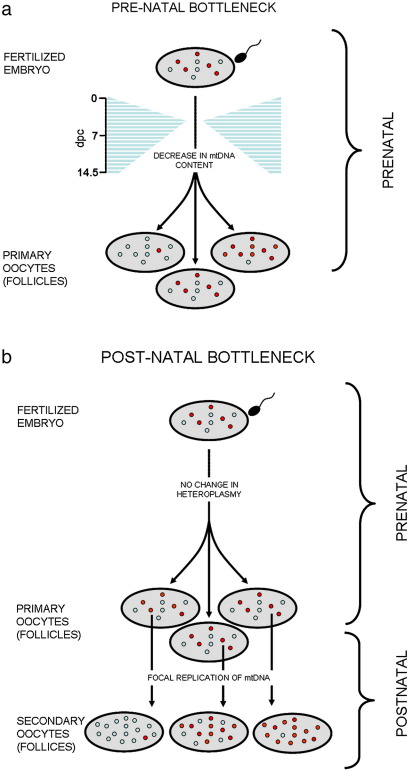
Models of the mitochondrial genetic bottleneck adapted from [52]. Schematic diagram showing a heteroplasmic fertilized oocyte (top), a model of the mitochondrial genetic bottleneck (middle) and subsequent oocytes (bottom). Blue circles = wild-type mtDNA. Red circles = mutated mtDNA. (a) Prenatal bottleneck. The variance in heteroplasmy levels arises during early embryo and germ-line development due to the random segregation of individual mtDNA molecules before the primary oocyte stage. This model is based on the actual amount of mtDNA measured within single cells as reported in [52] and [53]. Time scale shown on the left in days post coitus (dpc). (b) Postnatal bottleneck. The variance in heteroplasmy levels arises after birth due to the preferential replication of a subpopulation of mtDNA molecules as proposed in [53].

Using a different approach, Wai et al. have recently challenged the currently held notion that the genetic bottleneck occurs during embryonic oogenesis [54]. Using heteroplasmic mice expressing EGFP from a modified *Oct4* promoter they measured both mtDNA copy number and genotypic variance in both the post-implantation and post-natal stages of female development. During embryonic oogenesis, the primordial germ cells contained ∼ 280 copies of mtDNA at 8.5 dpc, rising to ∼ 2200 copies by 10.5 dpc. These results are consistent with those of Cree et al. (but not Cao et al. [52]), confirming that our direct measurement of the bottleneck was accurate [53]. By 14.5 dpc this had risen to ∼ 6000 copies of mtDNA per cell. These results suggest that there is still a far lower mtDNA copy number within the developing PGCs compared to fertilized oocytes (700 fold lower at 8.5 dpc, rising to 10–20 fold at 14.5 dpc). Using two nonparametric tests for equal variances, Wai et al. [54] did not observe a corresponding increase in genotypic variance over this same time point. However, when they studied post-natal development they noted a disparity in the genotypic variance between PGCs, oogonia and primary oocytes when compared to mature ovulated oocytes and primary oocytes within secondary follicles, implicating the post-natal expansion of mtDNA in the generation of varying heteroplasmy levels. Moreover, the authors used BrdU to label replicating mtDNA and staining using TFAM, mt-SSB and POLG, all markers of the mammalian nucleoid. They noted that only a subgroup of nucleoids was replicating in the primordial and primary follicles. They therefore suggested that this provides further evidence for selection of a random subset of nucleoids for replication in these cells (Fig. 1).

In conclusion, two independent studies agree that there is a significant reduction in mtDNA content within early PGCs, to a value of ∼ 200 copies per cell. The data of Wai et al. [54] directly implicates a post-natal mechanism in generating some of the increased variance between oocytes, but the absence of statistically significant increased variance between PGCs before birth does not mean that variation is not being generated at this time point. The only firm conclusion is that, based on a limited number of experiments, the variance did not change sufficiently to reject the null hypothesis of no variance on which the statistical tests were based. In other words, the results could simply reflect differences which fell below the detection threshold for statistical tests used to compare limited data sets. Direct measurements of heteroplasmy in large numbers of PGCs and oocytes at each stage of development are required to resolve this issue.

## 7 Conclusion and future perspectives

The molecular and cellular mechanisms of inheritance of mtDNA have been a source of intense speculation for decades, but recent technical advances have started to resolve some of the key issues in mice. It is now clear that selection operates during the transmission of some heteroplasmy mtDNA mutations in mice, but the mechanisms may lead to either the loss or accumulation of pathogenic mtDNA mutations. Although there does appear to be a restriction in mtDNA content early in the mouse germ cell lineage, direct experimental evidence is required to prove that this “bottleneck” makes a substantial contribution to the variation in heteroplasmy levels seen amongst the offspring of heteroplasmic female mice. The relative contribution of pre- and post-natal oocyte development in generating variation on heteroplasmy levels has yet to be established, and several major questions still remain. What genes control the genetic bottleneck? Is the bottleneck the same for all mtDNA mutations? How variable is the bottleneck between individuals? Does the same mechanism operate in humans? Can we manipulate the mechanism to prevent the transmission of pathogenic mtDNA mutations? The answers to these questions are just around the corner, providing hope for families transmitting these common but devastating genetic diseases.
